# Small Bowel Obstruction Secondary to Meckel's Diverticulum: A Rare Complication

**DOI:** 10.7759/cureus.72826

**Published:** 2024-11-01

**Authors:** Sima Patel, Ceri Gillett, Sanaa Elgaddal

**Affiliations:** 1 Surgery, New Cross Hospital, Wolverhampton, GBR; 2 General Surgery, Royal Stoke University Hospital, Stoke, GBR; 3 General and Colorectal Surgery, New Cross Hospital, Wolverhampton, GBR

**Keywords:** gastrointestinal obstruction, meckel's diverticulum, small bowel obstruction, small bowel resection, urgent laparotomy

## Abstract

Appendicitis is defined as an inflammation of the appendix and is one of the most common presentations to the general surgical team. The presence of right iliac fossa pain associated with a rise in inflammatory markers raises the suspicion of appendicitis in young, healthy patients. Colitis and bowel obstruction would be considered lower down on the list of differentials. Other common differentials to take into consideration include ovarian pathology and pelvic inflammatory disease in females and diverticulitis in the other population.

Investigation for these patients includes ultrasound scans, computed tomography, magnetic resonance imaging or diagnostic laparoscopy. Imaging modality can help narrow down differentials; however, diagnostic laparoscopy can prove to be fruitful when there is diagnostic uncertainty.

Meckel's diverticulum is commonly encountered within general surgery, more so within the paediatric population. Complications include bleeding, inflammation and obstruction, although this is less common in the adult population.

We present a rare case of small bowel obstruction with the transition point noted to be at the location of a Meckel's diverticulum masquerading as possible appendicitis.

## Introduction

Small bowel obstruction is described as a mechanical blockage of the bowel that prevents the normal passage of bowel contents through it [1]. There are many pathological processes that can lead to small bowel obstruction. In industrialised countries with advanced economies, adhesions are the leading cause of small bowel obstruction. Malignancy, hernias and inflammatory bowel diseases are also potential causes of small bowel obstruction [2].

During the embryonic stage, the omphalomesenteric duct connects the primitive midgut to the yolk sac. During normal development, the omphalomesenteric duct is obliterated and reabsorbed between the 7th week and 10th week [3]. Failure of the process can lead to a spectrum of omphalomesenteric duct anomalies, with Meckel’s diverticulum being the most common presentation of this omphalomesenteric duct remnant [3,4].

Most patients with a Meckel’s diverticulum remain asymptomatic, although complications can include haemorrhage (most common in the paediatric population), inflammation, intussusception, small bowel obstruction, perforation, diverticulitis and rarely neoplasm development [3].

We present a rare case of small bowel obstruction caused by an inflamed Meckel’s diverticulum.

## Case presentation

A 24-year-old female patient, with no significant past medical history, presented with a two-day history of migratory abdominal pain associated with nausea, vomiting and poor oral intake. The patient denied any significant gynaecological symptoms, bowels were opening as normal and there were no urinary symptoms.

On examination, the patient was clinically stable with no stigmata of disease. The patient's abdominal examination demonstrated a mildly distended, soft abdomen with maximum tenderness noted in the right lower quadrant on deep palpation. Urine analysis showed nil significant and urinary beta-human chorionic gonadotropin level (BHCG) was negative.

Blood tests on admission are shown in Table 1.

**Table 1:** Blood results of the patient on admission

Blood Test	Level Admission	Normal Range	Unit
Urea	5.9	2.5 – 7.8	mmol /L
Creatinine	87	50-98	mmol /L
Estimated glomerular filtration rate (eGFR)	80	>59	ml/min/1.73m^2^
Alkaline phosphatase (ALP)	108	30 - 130	IU/L
Alanine transaminase (ALT)	31	0-55	IU/L
Bilirubin	14	5-26	umol/L
Amylase	67	28 - 100	IU/L
Urinary HCG	<2.3	<2.3	IU/L
C-reactive protein	11	0.0 – 5.0	mg/L
Haemoglobin (Hb)	161	115 - 165	g/L
White cell count	14.90 x 10^9^	4.0 - 11.0	10^9 ^/L
Neutrophils	12.69 x 10^9^	2 – 7.5	10^9 ^/L
Platelets	455 x 10 ^9^	15- - 450	10^9 ^/L

A provisional diagnosis of possible appendicitis was made based on the biochemical findings and clinical examination. Initial management included intravenous antibiotics, intravenous fluids, and analgesia and the patient was kept nil by mouth. An ultrasound scan of the abdomen and pelvis was performed with view to confirm the pathology and reported as “thin-walled gallbladder, no gallstones. No bile duct dilatation. Trace fluid in the flanks. No appendiceal masses detected however numerous loops of fluid-filled, non-peristalsing bowel seen throughout”.

**Figure 1:**
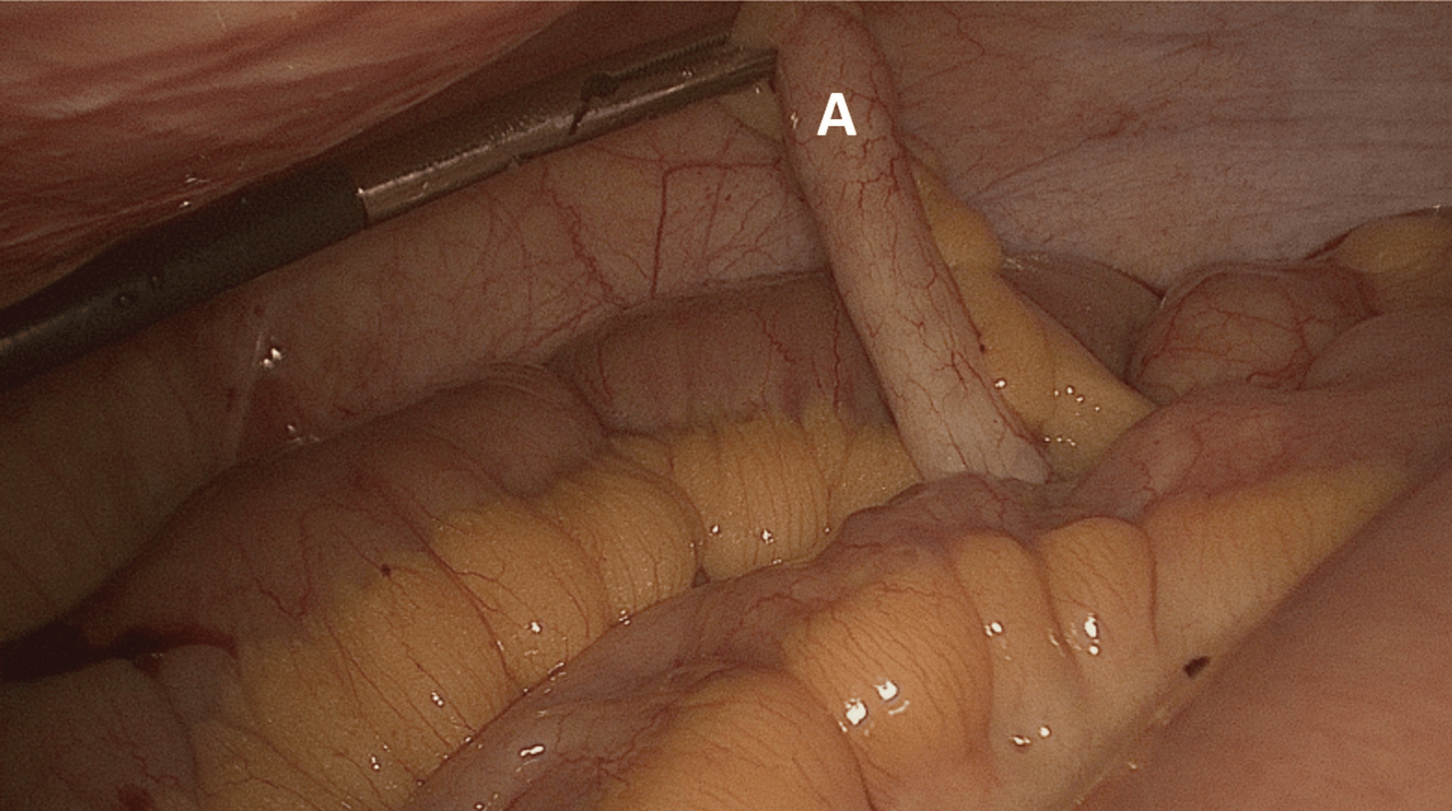
(A) Macroscopically normal looking appendix

**Figure 2:**
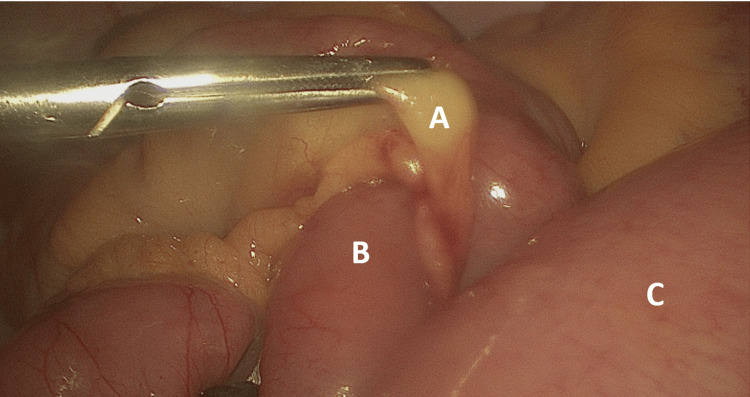
(A) Omental band adhesions with small bowel herniating through the defect (B). (C) Dilated small bowel loops distal to the point of obstruction

The patient underwent an emergency diagnostic laparoscopy. Intra-operative findings were as follows: macroscopically normal appendix (Figure 1), dilated and collapsed small bowel loops throughout the abdomen and a macroscopically inflamed Meckel’s diverticulum, measuring approximately 40 x 27 x 17mm with a band of omentum at the tip. The omental band was noted to be attached to an adjacent small bowel loop and there was another small bowel loop noted to be herniating through this created defect (Figure 2).

**Figure 3:**
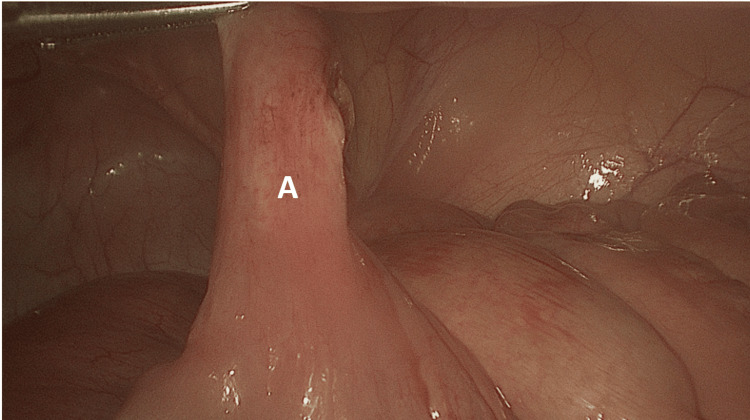
(A) Base of Meckel’s diverticulum showing its connection at the base to small bowel

**Figure 4:**
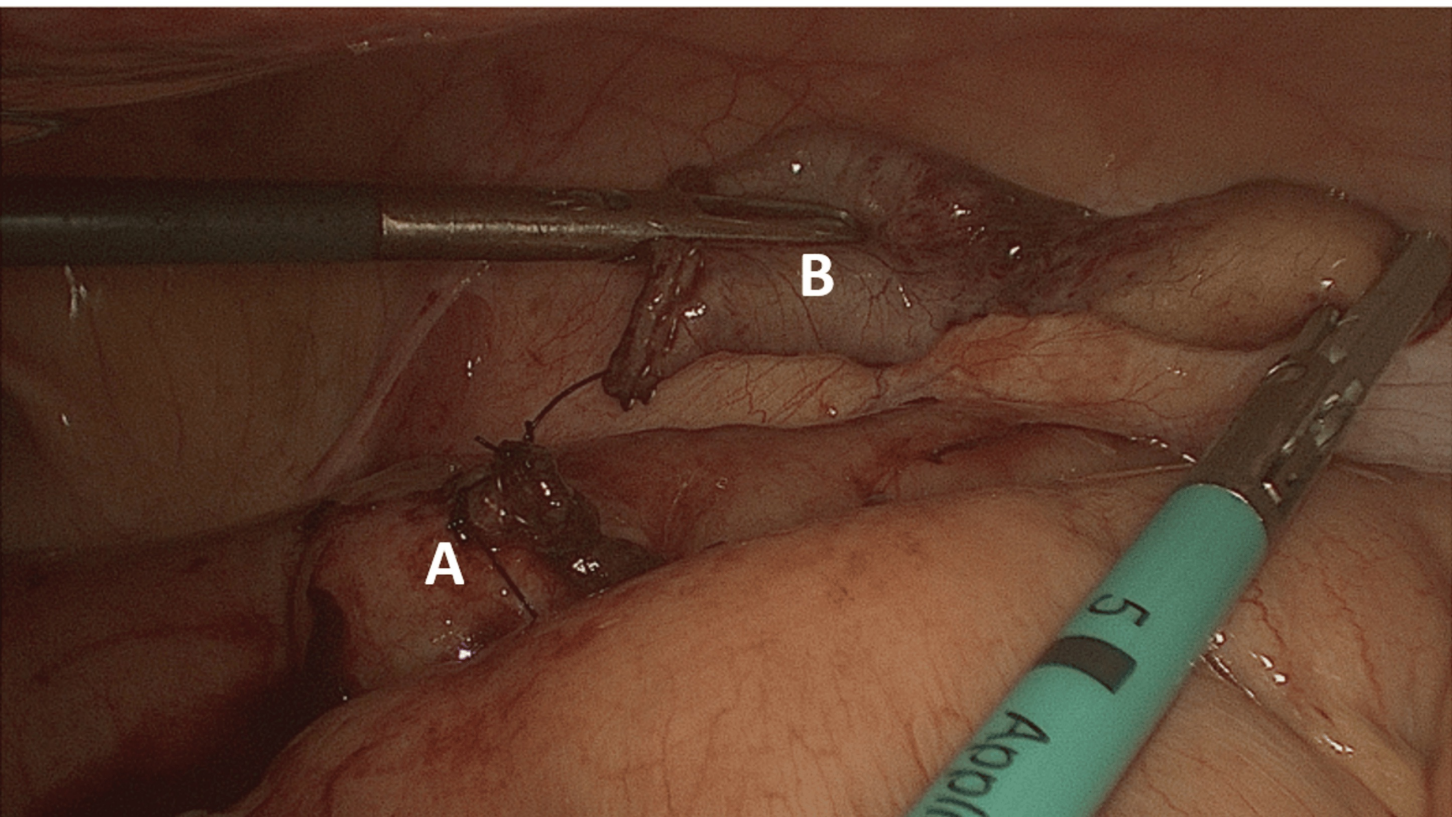
Resected Meckel’s diverticulum showing the staple line (A) and specimen (B)

Intra-operatively, the band adhesion was divided under direct vision, as shown in Figure 3, and Meckel’s diverticulum was resected with a 45mm Compact Articulating Endoscopic Linear Cutter ^TM^ (Ethicon Endo-Surgery, LLC, United Kingdom) with the base oversewn for haemostatic control (Figure 4).

The sample was sent for histology which confirmed the tissue to be small bowel with a diverticulum lined by typical small bowel mucosa. No ectopic tissue, inflammation, faecolith or malignancy was identified. Histology findings were in keeping with a Meckel’s diverticulum. The patient’s recovery was delayed due to prolonged ileus and ongoing antibiotic cover but was successfully discharged four days post operative intervention with outpatient follow-up.

## Discussion

With 50,000 acute appendicectomies performed annually in the United Kingdom, the presentation of right iliac fossa pain, nausea and the patient being generally unwell raise the suspicion of acute appendicitis; however, other clinical differentials to consider include acute gastritis, inflammatory bowel disease and obstruction [5]. In 1809, a German anatomist named Johann Friedrick Meckel described and officially named Meckel’s diverticulum, although reference to and identification of the diverticulum was initially identified by Wilhelm Fabry in 1598 [4,6].

Epidemiology and pathophysiology

It is estimated that there is a 2% prevalence of Meckel’s diverticulum within the general population. However, the exact prevalence is difficult to know due to many patients being asymptomatic and therefore not engaging with healthcare providers [7].

Although there is no clear familial connection in the development of Meckel’s diverticulum, risk factors for Meckel’s diverticulum and the development of symptoms include patients aged younger than 50 years old, male gender, diverticulum length greater than 2cm, ectopic tissue being present, broad-based diverticulum and fibrous bands attached to the diverticulum [7,8].

Embryology

In the embryonic stages, the midgut is connected to the yolk sac via the omphalomesenteric duct (vitelline duct) and this is the primary source of foetal nutrition during this embryonic period. Between the 5th week and 7th week of gestation, the omphalomesenteric duct is obliterated and reabsorbed and primary nutrition for the developing foetus is obtained from the placenta [4].

Blood supply to the omphalomesenteric duct is primarily from a pair of vitelline arteries. During the growth of the foetus, the left vitelline artery involutes while the right vitelline artery goes on to develop into the superior mesenteric artery, supplying the midgut of the bowel. This embryonic development explains why Meckel’s diverticulum obtains its blood supply directly from the mesentery of the midgut [4].

Failure of this process leads to a range of omphalomesenteric duct abnormalities, of which Meckel’s diverticulum is the most commonly encountered [2,4,9]. Another consequence due to failure of this step is fistula development between the ileum and the umbilicus (the entire duct is patent). This creates a direct connection between the umbilicus and ileum which may result in seepage of bilious intestinal contents from the umbilicus [2,4]. A further consequence includes the formation of an umbilicus sinus (closed-end duct when the umbilic end of the duct is not closed off) [2]. A fibrous cord can develop on both sides of the omphalomesenteric duct, leaving a cyst at the mid-point (vitelline cyst) [4]. A fibrous cord can also develop between the umbilicus and ileum which may create a fixed point around which bowel loops may become tangled which can cause bowel obstruction [2,4].

The cells lining the vitelline duct are pluripotent cells and are therefore capable of differentiating into any cell type found within the human body [10]. Within a Meckel’s diverticulum, heterotopic tissue can be found with common subtypes including gastric and pancreatic tissue [10,11].

Signs and symptoms

Although most patients with Meckel’s diverticulum remain asymptomatic, 4 to 16% of patients become symptomatic [1,8]. The most common presentation of Meckel’s diverticulum is painless rectal bleeding [2,7-9], largely due to ectopic tissue. In normal development, the duodenum is able to secrete bicarbonate to neutralise gastric contents. In ectopic gastric tissue, the gastric mucosa secretes an acid that can lead to ulceration of mucosa and therefore painless bleeding. Patients may also present with features of bowel obstruction due to a combination of factors, as outlined earlier in the article. Other signs include small bowel contents seeping from the umbilicus (patent omphalomesenteric duct) or purulent discharge from the umbilicus (umbilical sinus) [2,7-9].

Finally, as previously flagged, patients with Meckel’s diverticulum may be completely asymptomatic and therefore do not need to engage with healthcare providers. Therefore, Meckel’s diverticulum may also be picked up incidentally when investigating other symptoms [8].

Management of Meckel’s diverticulum

While Meckel’s diverticulum can be an incidental finding, an awareness of management options and evidence to support these options are vital in order to provide the best care for patients. Risk factors that may increase the likelihood of the patient requiring a resection for symptomatic Meckel’s diverticulum include patients younger than 50 years old, male patients, diverticulum longer than 2 cm and detection of abnormal features inside the diverticulum [12-14].

Numerous studies have concluded that a large number of resections of Meckel’s diverticulum would need to be performed in order to prevent death [12,13]. Research has also concluded that resection of incidentally found Meckel’s diverticulum was not advised due to resection not outweighing both the morbidity and mortality associated with surgical intervention [13].

The definitive treatment option for symptomatic Meckel’s diverticulum is surgery, either laparoscopic, laparoscopic-assisted or open resection of the small bowel with the extent of resection guided by the complexity of the case and intra-operative findings [8,12-14].

## Conclusions

The presence of a Meckel’s diverticulum is not limited to the paediatric population and although rare, can lead to complications in the adult population.

Although small bowel obstruction secondary to an omental band from a Meckel’s diverticulum is a rare complication, it should be considered as a potential differential in patients who present with small bowel obstruction, especially in patients who have not had any previous intra-abdominal surgery. 
